# Rapid and Highly Efficient Generation of Induced Pluripotent Stem Cells from Human Umbilical Vein Endothelial Cells

**DOI:** 10.1371/journal.pone.0019743

**Published:** 2011-05-16

**Authors:** Athanasia D. Panopoulos, Sergio Ruiz, Fei Yi, Aída Herrerías, Erika M. Batchelder, Juan Carlos Izpisua Belmonte

**Affiliations:** 1 Gene Expression Laboratory, The Salk Institute for Biological Studies, La Jolla, California, United States of America; 2 Center of Regenerative Medicine in Barcelona, Barcelona, Spain; City of Hope National Medical Center and Beckman Research Institute, United States of America

## Abstract

The ability to induce somatic cells to pluripotency by ectopic expression of defined transcription factors (e.g. *KLF-4*, *OCT4*, *SOX2*, *c-MYC*, or KOSM) has transformed the future of regenerative medicine. Here we report somatic cell reprogramming of human umbilical vein endothelial cells (HUVECs), yielding induced pluripotent stem (iPS) cells with the fastest kinetics, and one of the highest reprogramming efficiencies for a human somatic cell to date. HUVEC-derived iPS (Huv-iPS) cell colonies appeared as early as 6 days after a single KOSM infection, and were generated with a 2.5–3% reprogramming efficiency. Furthermore, when HUVEC reprogramming was performed under hypoxic conditions in the presence of a TGF-beta family signaling inhibitor, colony formation increased an additional ∼2.5-fold over standard conditions. Huv-iPS cells were indistinguishable from human embryonic stem (ES) cells with regards to morphology, pluripotent marker expression, and their ability to generate all embryonic germ layers *in vitro* and *in vivo*. The high efficiency and rapid kinetics of Huv-iPS cell formation, coupled with the ease by which HUVECs can be collected, expanded and stored, make these cells an attractive somatic source for therapeutic application, and for studying the reprogramming process.

## Introduction

Seminal studies demonstrated that fibroblasts could be reprogrammed to pluripotency by ectopic expression of defined factors [1]–[3]. Somatic cell reprogramming has now been performed with numerous somatic sources with variable kinetics and efficiencies [4], [5]. Among these, recent reports have demonstrated that iPS cells can be generated from human peripheral blood samples, advancing practical methods of obtaining patient-specific iPS cells [6]–[8]. However, while the accessibility of this somatic cell source provides an advantage, reprogramming blood samples is not an efficient (∼0.001–0.1%) or rapid (∼1 month) process [6]–[8]. More importantly, primary blood samples cannot be continually passaged or easily manipulated, resulting in limited flexibility to generate iPS cells.

In contrast, human fibroblasts are amenable to culture manipulations, but are also inefficient (<0.01%) and slow (∼1 month) in undergoing reprogramming [2], [3]. We therefore sought to find a more practical cell type that could be readily isolated and expanded, yet could reprogram quickly and efficiently. Here we report the rapid reprogramming of HUVECs, with an efficiency approximately 300-fold higher than human fibroblasts [2], [3]. The methods by which HUVECs can be readily obtained in large quantities without purification steps [9], coupled with their fast and efficient rate of reprogramming, makes this somatic cell source practical for therapeutic application, and for studying the mechanisms governing reprogramming.

## Methods

### iPS cell generation

Keratinocytes or HUVECs (Lonza) were infected at similar passage (generally at p2 or p3) with equivalent ratios of retroviruses encoding KOSM by spinfection at 800 g for 1 hour at RT in the presence of polybrene (8 µg/ml). Cells were replated onto MEFs (Millipore) in their respective media, and switched to ES cell medium for iPS cell colony formation. Resulting iPS cell colonies were either manually picked for iPS cell line derivation (∼10–12 days after infection), or stained for Nanog as described (∼14–20 days after infection, to enable iPS cell colony formation from keratinocyte controls). Reprogramming efficiencies were then determined by calculating the number of Nanog positive colonies as a percentage of GFP positive cells. For reprogramming experiments performed in hypoxic conditions, cells were placed in 5% 0_2_ incubators 4 days after the initial infection (i.e. when the cells were switched to ES cell medium) where they remained for the duration of the assay. For TGF-beta family signaling inhibitor reprogramming experiments, cells were treated daily with 10 µM SB431532 (Sigma) from day 4 to day 10 after the initial infection, and then were treated with ES cell media without SB431532 for the duration of the assay.

### Cell lines and culture

Human neonatal keratinocytes (Lonza) and HUVECs (Lonza) were grown according to manufacturer's recommendations. 293T cells (ATCC) were cultured in DMEM (Invitrogen) containing 10% fetal calf serum (FCS). Human H1 or H9 ES cell lines (Wicell) were cultured as previously described [10]–[12]. iPS cell lines obtained from keratinocytes [13] (KiPS4F2, KiPS4FA, KiPS4FB), astrocytes [14] (ASTiPS4F5) or fibroblasts [15] (FiPS4F5) were used as controls, and were all fully characterized using similar methodologies and criteria as described herein.

### Plasmids and virus preparation

The following moloney murine leukemia virus-based retroviral vectors were obtained from Addgene: pMXs-hOCT4, pMXs-hSOX2, pMXs-hKLF4 and pMXs-hc-Myc (plasmids 17217, 17218, 17219 and 17220 respectively). pMXs-eGFP was kindly provided by Dr. Teruhisa Kawamura (Gene Expression Laboratory, The Salk Institute, La Jolla, CA). Packaging plasmids (pCMV-gag-pol-PA and pCMV-VSVg) were kindly provided by Dr. Gerald Pao, Laboratory of Genetics, The Salk Institute, La Jolla, CA. Retrovirus was collected 24 hours following cotransfection of plasmids in 293T cells using Lipofectamine (Invitrogen) in accordance with manufacturer's recommendations.

### Immunofluorescence and immunohistochemistry

For the immunohistochemical detection of Nanog, cells were first fixed with 4% formaldehyde in PBS for 15 minutes at room temperature (RT). Following washing in PBS, cells were incubated in 0.4% Triton-X100/PBS at RT for 10 minutes. A rabbit anti-human Nanog antibody (1∶500, Abcam) was diluted in PBS containing 1% BSA (PBS/BSA) and was used for overnight incubation at 4°C. Cells were then washed in PBS/BSA, and incubated with a secondary biotin-conjugated anti-rabbit antibody (1∶2000) for an additional 2 hours at RT. After washing in PBS/BSA, cells were incubated at RT with streptavidin-HRP (Vector, 1∶1000) for 2 hours, and a DAB substrate kit for peroxidase (Vector, SK-4100) was used to develop the staining.

For standard immunofluorescence, cells were fixed and permeabilized as described, followed by blocking in PBS/BSA containing 5% FCS for 1 hour at RT. Antibodies to FoxA2 (R&D), Tuj-1 (Covance), alpha smooth muscle actin (ASMA, Sigma) were diluted in PBS/BSA and used in overnight incubations at 4°C, followed by incubation with fluorescently-conjugated secondary antibodies (AlexaFluor 488 or AlexaFluor 568, Invitrogen) for 2 hours at RT. 4,6-Diamidino-2-phenylindole (DAPI) was used to visualize nuclei at a concentration of 10 µg/ml in PBS. Additional immunofluorescence, and hematoxylin and eosin staining, was performed as previously described [13].

### Flow cytometry

Cells were collected with TrypLE (Invitrogen), resuspended in PBS containing 1% BSA (PBS/BSA) and labeled with fluorescently conjugated antibodies to Tra-1-60 (BD Pharmingen), Tra-1-81 (Stemgent), SSEA-4 (BD Pharmingen), CD34 (BD Pharmingen), CD45 (BD Pharmingen), CD31 (BD Pharmingen), or the appropriate isotype controls. Samples were analyzed by flow cytometry on a FACScan (Becton-Dickinson), and figures generated using FlowJo software (TreeStar Inc).

### Real-time PCR

To determine gene expression levels, total RNA was first isolated using Trizol Reagent (Invitrogen), and reverse transcribed using the SuperScript II Reverse Transcriptase kit (Invitrogen), according to the manufacturer's recommendations. Real-time PCR analysis was performed using the SYBR-Green PCR Master mix (Applied Biosystems). The expression values of individual genes were normalized to *GAPDH*, and are shown relative to control samples as indicated. See Table 1 for a complete list of primers.

**Table 1:** List of primers.

Primer	Sequence (5′ to 3′)	Application
OCT4 total-F	GGAGGAAGCTGACAACAATGAAA	qPCR
OCT4 total-R	GGCCTGCACGAGGGTTT	qPCR
SOX2 total-F	TGCGAGCGCTGCACAT	qPCR
SOX2 total-R	TCATGAGCGTCTTGGTTTTCC	qPCR
KLF4 total-F	CGAACCCACACAGGTGAGAA	qPCR
KLF4 total-R	GAGCGGGCGGCGAATTTCCAT	qPCR
c-MYC total-F	AGGGTCAAGTTGGACAGTGTCA	qPCR
c-MYC total-R	TGGTCGATTTTCGGTTGTTG	qPCR
OCT4 end-F	GGGTTTTTGGGATTAAGTTCTTCA	qPCR
OCT4 end-R	GCCCCCACCCTTTGTGTT	qPCR
SOX2 end-F	CAAAAATGGCCATGCAGGTT	qPCR
SOX2 end-R	AGTTGGGATCGAACAAAAGCTATT	qPCR
KLF4 end-F	AGCCTAATTGATGGTGCTTGGT	qPCR
KLF4 end-R	TTGAAAACTTTGGCTTCCTTGTT	qPCR
c-MYC end-F	CGGGCGGGCACTTTG	qPCR
c-MYC end-R	GGAGAGTCGCGTCCTTGCT	qPCR
GAPDH-F	GGACTCATGACCACAGTCCATGCC	qPCR
GAPDH-R	TCAGGGATGACCTTGCCCACAG	qPCR
OCT4 genomic-F	AGCGATCAAGCAGCGACTAT	qPCR
OCT4 genomic-R	GTGAAGTGAGGGCTCCCATA	qPCR
SOX2 genomic-F	AACCCCAAGATGCACAACTC	qPCR
SOX2 genomic-R	GCTTAGCCTCGTCGATGAAC	qPCR
KLF4 genomic-F	GTCTCTTCGTGCACCCACTT	qPCR
KLF4 genomic-R	TGCTCAGCACTTCCTCAAGA	qPCR
c-MYC genomic-F	CCCTCAACGTTAGCTTCACC	qPCR
c-MYC genomic-R	CAGCAGCTCGAATTTCTTCC	qPCR
GAPDH genomic-F	ACCCAGAAGACTGTGGATGG	qPCR
GAPDH genomic-R	TTCAGCTCAGGGATGACCTT	qPCR

For the stem cell array analysis between ES cells and the Huv-iPS cell lines, real-time PCR of a selected number of genes based on the Human Stem RT2 Cell Array (SuperArray Bioscience Corporation) was performed. These genes differentially discriminate between pluripotent and somatic cell types. For a detailed description of the genes and primer sets used for this analysis see Ruiz et al [14].

In order to determine the copy numbers of transgenes introduced by reprogramming, a quantitative real-time PCR method was developed to detect both endogenous and transgenic numbers of four reprogramming factors (*OCT4*, *SOX2*, *KLF-4*, *c-MYC*). Briefly, primer sets specifically detecting the coding sequence within a single exon were designed for each reprogramming factor, and tested for both ES cell genomic DNA (gDNA) and reprogramming retroviral vectors. High quality gDNA samples were prepared using QIAGEN DNeasy Blood & Tissue Kit (QIAGEN), and measured by NanoDrop 8000 Spectrophotometer (Thermo Scientific). For each reaction, 10 ng gDNA from each sample were run in triplicate along with four points of ES cell gDNA standard curve templates made by 10-fold serial dilutions (from 100 ng to 0.1 ng) to ensure adequate amplification efficiency (>90%). Levels of each reprogramming factor were normalized to *GAPDH* for each sample, and calculated relative to the endogenous levels in ES cells (2 copies of each factor per genome). The results were presented as means +/− standard deviations of both endogenous and transgenic copy numbers. All PCR reactions were performed using the SYBR-Green PCR Master mix on the ViiA 7 Real-time PCR system (Applied Biosystems) in accordance to the manufacturer's instructions, and were repeated three times. See Table 1 for a complete list of primers.

### *In vitro* differentiation

For embryoid body (EB) differentiation, ES or Huv-iPS cell colonies growing on MEFs (Millipore) were loosely detached by dispase treatment, washed and resuspended in EB media (DMEM/F12 containing 10% FCS (Atlanta Biologicals), 0.5 mM L-glutamine, 0.1 mM non-essential amino acids and 55 µM β-mercaptoethanol). EBs were maintained on low attachment plates and replenished daily with fresh EB media. After 4 days, EBs were plated on gelatin-coated plates, allowed to differentiate for another 10 days in EB media, fixed and stained as described.

### Teratoma assay and karyotype analysis

To test for teratoma formation, iPS cell lines were injected into severe combined immunodeficient mice (NOD.Cg-*Prkdc^scid^ Il2rg^tm1Wjl^*/SzJ; Jackson Laboratories). Briefly, ∼10^6^ iPS cells in ∼50 µL of ES cell medium were injected into the kidney capsule or testes of anesthetized mice. Mice were then monitored for formation of teratomas, and euthanized ∼6–12 weeks after injection. Collected teratomas were analyzed by immunofluorescence or hematoxylin and eosin staining as previously described [13]. All mouse experimental procedures were performed and approved (accepted protocol number 08–025) by The Salk Institute Institutional Animal Care and Use Committee (IACUC). All Huv-iPS cell lines were karyotyped by Wicell.

### Statistical analysis

Results are shown as mean values ± standard deviation (SD) or standard error of the mean (SEM) as indicated. The values obtained for the stem cell array were analyzed using the Pearson correlation coefficient as a measure of similarity. Remaining statistics were performed using unpaired two-tailed Student's *t*-tests. *P* values<0.05 were considered statistically significant.

## Results

**Figure 1:**
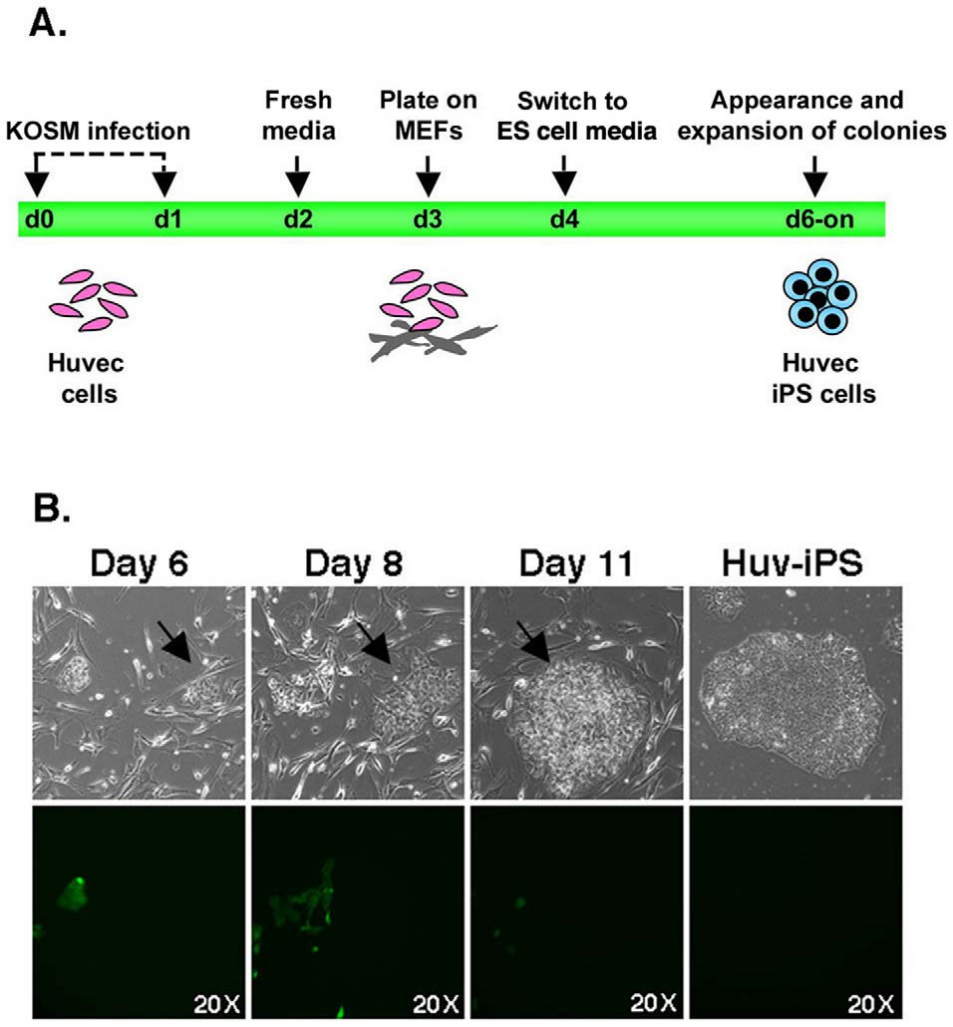
Derivation of induced pluripotent cells from HUVECs. HUVECs were retrovirally infected with *KLF-4*, *OCT4*, *SOX2* and *c-MYC* (KOSM) to generate induced pluripotent stem cells (Huv-iPS4F). (A) Schematic representation of the experimental strategy used to reprogram HUVECs. (B) Infected HUVECs were plated onto mouse embryonic fibroblasts (MEFs) and colony formation assessed. Retroviral transduction of GFP was included to measure infection efficiency, and monitor silencing of transgenes during reprogramming. Note the appearance of GFP negative colonies with an ES cell-like morphology as early as 6 days after infection, as demonstrated by tracking an individual colony (black arrow) from day 6 through day 11. An example of an established Huv-iPS cell line grown in feeder-free conditions is shown on the right. All images were acquired with a standard microscope using a 20× objective; all fluorescent images shown were acquired with the same exposure time.

**Figure 2:**
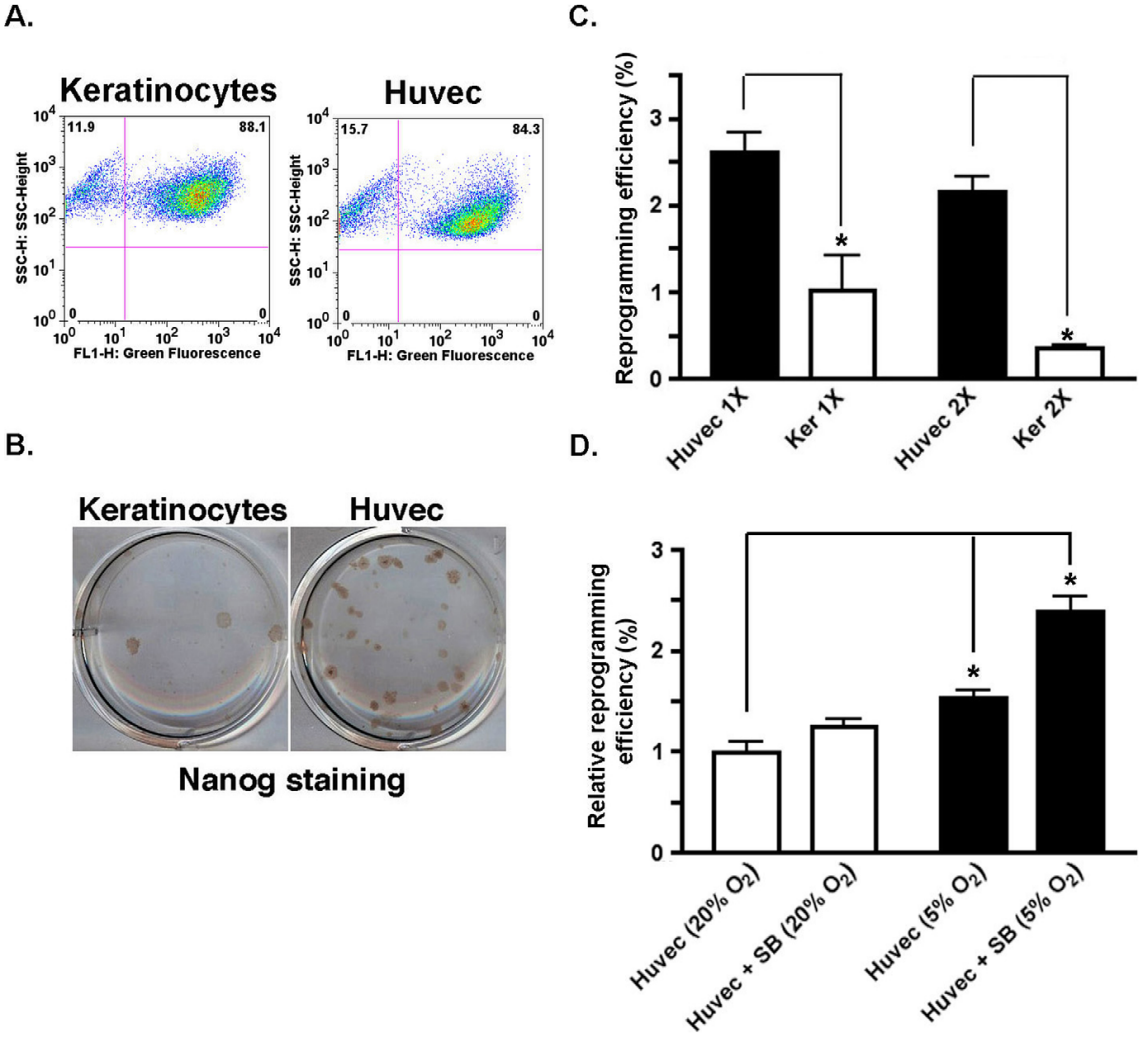
Reprogramming of HUVECs is highly efficient. (A) HUVECs and keratinocytes were infected in parallel with retroviruses encoding KOSM and GFP. Shown are representative histograms of GFP expression for each cell type 3 days after infection, as assessed by flow cytometry. (B) Equivalent numbers of GFP positive cells were plated on MEFs, and a representative example of immunohistochemical staining (of an individual well from a 6-well plate) for Nanog of the resulting colonies is shown. MEF feeder layers serve as an internal negative control for Nanog staining. (C) HUVECs and keratinocytes (Ker) were infected in parallel (a single infection, 1X, or two infections, 2X) plated, and stained for Nanog. Nanog positive colonies were numerated and plotted as a percentage of GFP positive cells, indicative of reprogramming efficiency. Results were quantified from triplicate samples, and are representative of at least three independent experiments. Error bars depict the standard error mean (SEM). (D) Equivalent numbers of KOSM-infected HUVECs (1X infection) were plated and placed in incubators containing 20% O_2_ (standard conditions) or 5% 0_2_ (hypoxic conditions) in the presence or absence of the TGF-beta family signaling inhibitor SB431532 (SB). The reprogramming efficiencies relative to controls are shown. Results were quantified from triplicate samples, and are representative of two independent experiments. Error bars depict the SEM. *P<0.05.

HUVECs were transduced with retroviruses encoding KOSM to induce somatic cell reprogramming (Figure 1A). Retroviral infections with GFP were included to assess infection efficiency, and to monitor transgene silencing [16]. We observed the appearance of colonies with an ES cell-like morphology as early as 6 days after two serial KOSM infections (Figure 1B). In several cases, these colonies were GFP negative, correlating with transgene silencing (Figure 1B). We next tested if a single infection would be sufficient to elicit the production of iPS cells. To assess the efficiency of HUVEC reprogramming, we performed parallel infection experiments with keratinocytes, a human somatic cell type with one of the highest reported reprogramming efficiencies to date [13]. HUVECs and keratinocytes were infected in parallel with retroviruses encoding KOSM and GFP on day 0 (1X), or day 0 and day 1 (2X), equivalent numbers of GFP positive cells plated, and resulting colonies stained for Nanog as an initial measure of pluripotency (Figure 2A, 2B). We routinely observed >80% transduction efficiency for all conditions (Figure 2A). Following a single KOSM infection, HUVECs displayed a 2.5–3% reprogramming efficiency, whereas keratinocytes demonstrated an approximate 1% reprogramming efficiency, in agreement with our previous findings (Figure 2C) [13]. Interestingly, two serial KOSM infections decreased reprogramming efficiencies for both cell types, although more strikingly for keratinocytes, and resulted in a more substantial efficiency difference between HUVECs and keratinocytes (1X = 2.5–3 fold difference vs. 2X = 7–8 fold difference, respectively; Figure 2C). These results indicate that the number of infections should be taken into account when determining reprogramming efficiencies, and suggest that the balance of viral incorporation and tolerance to infection varies for somatic cell types. Of note, HUVECs that had undergone additional freeze/thaws before infection, or had been passaged repeatedly (e.g. 7–8 passages), still generated the high reprogramming efficiencies indicated (Figure 2C).

Previous studies have demonstrated that hypoxia or inhibition of TGF-beta family signaling enhances iPS cell generation [17]–[19]. We next tested each of these conditions, alone or in combination, in HUVEC-mediated colony formation. Performing reprogramming under hypoxic conditions was sufficient to increase the reprogramming efficiency compared to controls grown in standard 20% O_2_ conditions (Figure 2D). However, treatment with the TGF-beta family signaling inhibitor SB431532 in combination with hypoxic conditions further increased reprogramming ∼2.5-fold over controls (Figure 2D).

**Figure 3:**
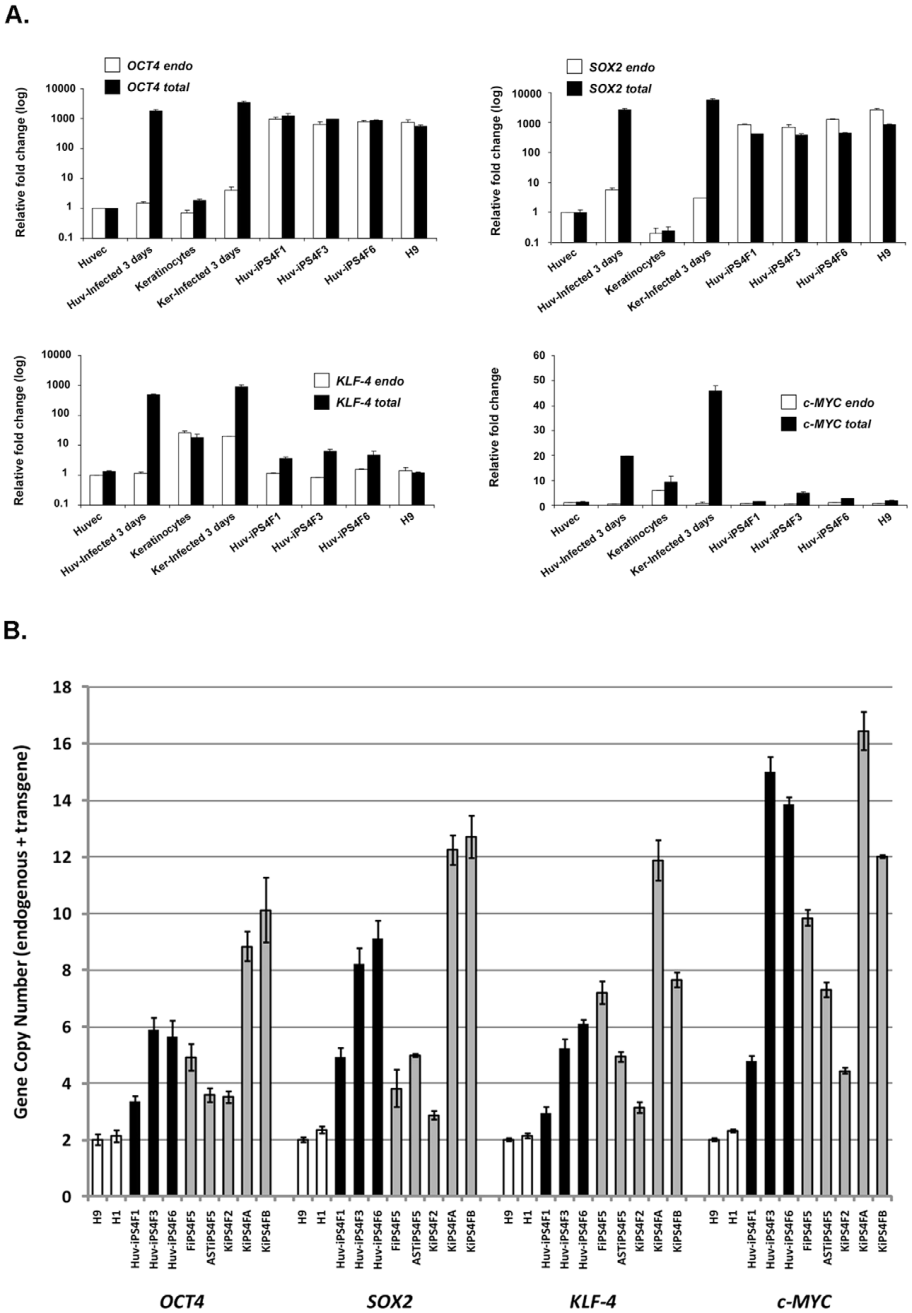
Reprogramming factor expression and transgene integration in Huv-iPS cells. (A) Gene expression levels of the endogenous and total levels of *OCT4*, *SOX2*, *KLF-4* and *c-MYC* in Huv-iPS cell lines, ES cell controls (H1 and H9 cell lines), somatic cells (keratinocytes and HUVECs) or somatic cells 3 days after KOSM infection. Individual real-time PCR reactions were normalized to *GAPDH*, and plotted relative to the expression level in HUVECs. Data are shown as the relative averages ± standard deviation (SD). (B) Copy numbers of the four reprogramming factor transgenes (*OCT4*, *SOX2*, *KLF-4* and *c-MYC*) were determined in Huv-iPS cell lines by quantitative real-time PCR as described in Methods. Data were presented as the copy number of both endogenous gene (2 copies/genome) and transgene (the portion higher than 2 copies/genome) for each reprogramming factor. Two human ES cell lines (H9, H1; white bars) were used as negative controls for transgenes. The copy numbers of transgenes in three Huv-iPS cell lines (Huv-iPS4F1, Huv-iPS4F3, Huv-iPS4F6; black bars) were compared to five other characterized iPS cell lines (gray bars) obtained from fibroblasts (FiPS4F5), astrocytes (ASTiPS4F5) or keratinocytes (KiPS4F2, KiPS4FA, KiPS4FB). Data are shown as the relative averages ± standard deviation (SD).

To characterize HUVEC-generated colonies, we manually picked ∼12 GFP negative colonies 10–12 days after KOSM infection, and randomly chose three lines (Huv-iPS4F1, Huv-iPS4F3, Huv-iPS4F6) for full characterization. We first evaluated the expression of the reprogramming factors, following the initial infection, as well as in the established Huv-iPS cell lines generated. Expression of *OCT4*, *SOX2*, *KLF-4* and *c-MYC* was induced at similar levels following 3 days of infection for both HUVECs and keratinocytes (Figure 3A). Individual Huv-iPS cell lines also demonstrated endogenous *OCT4*, *SOX2*, *KLF-4* and *c-MYC* gene expression levels that were similar to ES cell controls, and to the total (endogenous+transgene) expression levels for each gene (Figure 3A). Although this is indicative of strong transgene silencing, minor contribution from transgenes to the total expression of *KLF-4* (each line) or *c-MYC* (Huv-iPS4F3 cell line) was observed (Figure 3A). Furthermore, Huv-iPS cells showed transgene copy numbers at comparable levels to other iPS cell lines that had been generated using the same retroviral approach, but from less efficient somatic sources such as fibroblasts (FiPS4F5), astrocytes (ASTiPS4F5), and keratinocytes (KiPS4F2, KiPS4FA, KiPS4FB) (Figure 3B) [13]–[15]. Thus, the higher efficiency of HUVECs to generate iPS cells is not likely due to differences in infection efficiency or transgene integration, but to other as of yet undetermined mechanisms of inducing pluripotency.

**Figure 4:**
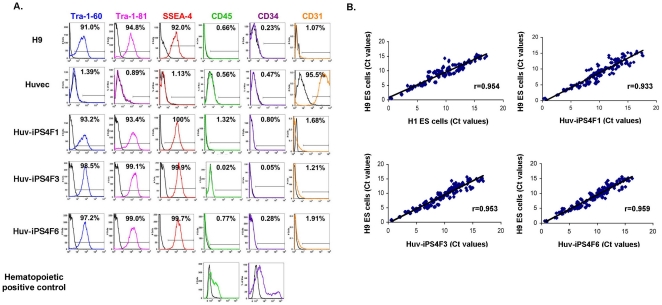
Huv-iPS cells express pluripotent markers. (A) Flow cytometry analysis for pluripotency (Tra-1-60, Tra-1-81, SSEA-4), hematopoietic (CD45, CD34) or endothelial (CD31) markers as indicated, for all Huv-iPS cell lines, and the appropriate positive controls (H9 ES cells, hematopoietic cells, or HUVECs, respectively). Percentages were determined relative to the appropriate isotype control (black lines) for each cell type. (B) Ct values obtained from real-time PCR analysis of a defined set of genes (see Methods) were normalized to *GAPDH* expression, and plotted to generate a graphical representation of the similarity between the different cell lines as indicated. r = Pearson coefficient.

We next evaluated pluripotency markers of each Huv-iPS cell line at the protein level. Cell surface protein marker analysis demonstrated that Huv-iPS cells expressed the pluripotent markers Tra-1-60, Tra1-81 and SSEA-4, and had lost expression of the endothelial marker CD31. Furthermore, the parental HUVEC populations were negative for CD45 and CD34, ruling out the contribution of any possible residual hematopoietic cells obtained from HUVEC preparations in the high reprogramming efficiencies observed (Figure 4A). To further assess the overall profile of Huv-iPS cell lines relative to ES cells, we analyzed the expression of several genes involved in various aspects of stem cell biology (see Methods). As shown in Figure 4B, using the Pearson correlation coefficient to measure the distance between the different sets of values, individual Huv-iPS cell lines had stem cell gene expression profiles that were as similar to ES cell controls as individual ES cell lines were to one another.

**Figure 5:**
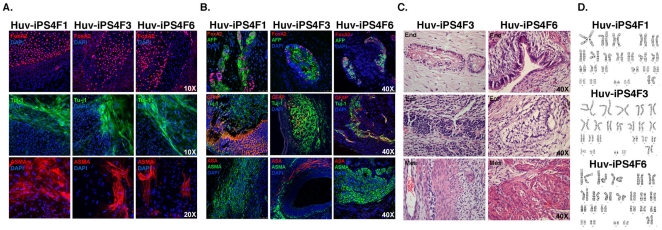
Huv-iPS cell lines demonstrate pluripotency *in vitro* and *in vivo*. (A) Huv-iPS cell lines were used in embryoid body (EB)-mediated differentiation assays, and stained by immunofluorescence for endodermal (FoxA2), ectodermal (Tuj-1), or mesodermal (alpha smooth muscle actin (ASMA)) markers representing each embryonic germ layer. 4,6-Diamidino-2-phenylindole (DAPI) staining shows nuclei. (B–C) Huv-iPS cell lines were injected into immunocompromised mice and analyzed for teratoma formation. Resulting teratomas were analyzed for tissues representing each of the three embryonic germ layers by (B) fluorescent imaging (endodermal markers FoxA2 and α-fetoprotein (AFP), upper panels; ectodermal markers GFAP and TuJ-1, middle panels; mesodermal markers alpha sarcomeric actin (ASA) and alpha smooth muscle actin (ASMA), lower panels; nuclei are stained with DAPI or by (C) hematoxlin and eosin staining (endoderm, upper panels; ectoderm, middle panels; or mesoderm, lower panels). All images for individual lines were obtained from a single tumor, and were acquired using a 40× objective. (D) Karyotype analysis demonstrating that Huv-iPS cell lines maintain normal chromosomal integrity.

As a final stringent analysis of Huv-iPS cell pluripotency, we evaluated the potential of each Huv-iPS cell line to differentiate into the three embryonic germ layers *in vitro* and *in vivo*. Immunofluorescence analysis of embryoid bodies differentiated from Huv-iPS cells showed the presence of markers for endoderm, ectoderm and mesoderm lineages (Figure 5A). Injection of Huv-iPS cell lines into immunocompromised mice produced teratomas, which contained tissues from all three embryonic germ layers (Figure 5B, 5C). Lastly, Huv-iPS cell lines displayed a normal karyotype (Figure 5D), and have been maintained in feeder-free conditions for over 40 passages. These collective results demonstrate the successful reprogramming of HUVECs into iPS cells, with the fastest kinetics and one of the highest efficiencies reported for any human somatic cell to date.

## Discussion

Our findings demonstrating rapid and highly efficient reprogramming of HUVECs are in contrast to a previous report, which showed that KOSM infection of HUVECs generated iPS cell colonies after more than two weeks, with a reprogramming efficiency that was 100-fold lower (∼0.03%) [20]. Very recent studies also demonstrated that iPS cells could be generated from HUVECs at <0.03% efficiency. However, in these reports reprogramming was performed using only SOX2 and OCT4 [21], or OCT4 and a combination of chemical compounds [22]; thus, the use of fewer factors are likely contributing to the lower reprogramming efficiencies and delayed kinetics observed in these instances [21], [22].

Although the reasons for some of these discrepancies remain unclear, variations in somatic cell sources, virus quality and infection protocols are known variables in reprogramming [16]. However, we have tested various HUVEC lots and consistently found reprogramming efficiency to be ∼2.5–3% with initial colony appearance ∼day 6, and thus it is unlikely that the source of HUVECs is causing these differences (Figure 2C). Additionally, we performed parallel transduction experiments with human keratinocytes and fibroblasts, and found that the observed reprogramming efficiencies and kinetics correlated with what has been previously reported in the literature (Figure 2C, data not shown) [2], [3], [13]. Thus, these collective data indicate that our reprogramming experiments are accurately assessing the reprogramming capabilities of HUVECs.

Our laboratory and others have reported the generation of iPS cells from human cord blood [23], [24], which provides the advantage of an available banked HLA-typed somatic cell source for reprogramming. Furthermore, iPS cells obtained from embryonic somatic sources have been shown to be safer than those obtained from adult cells [25], which have been subjected to mutagenic events during aging. HUVECs are isolated from newborn's umbilical cord with no risk to the donor, can be rapidly prepared without purification steps, and stored in large quantities [9]. Thus, HUVECs could be collected by cord blood banks, to serve as an alternative HLA-typed reprogramming source, since a reasonable amount of HLA-typed iPS cell lines could provide a beneficial match for a considerable percentage of the population [26], [27]. This would also enable the reserve of valuable cord blood samples for use in bone marrow transplantation. The rapid and efficient generation of iPS cells from HUVECs could also provide an important tool to discern the mechanisms governing reprogramming. These combined reasons make HUVECs an attractive somatic source for therapeutic application, and for studying the reprogramming process.

## References

[1] Takahashi K, Yamanaka S (2006). Induction of pluripotent stem cells from mouse embryonic and
adult fibroblast cultures by defined factors.. Cell.

[2] Takahashi K, Tanabe K, Ohnuki M, Narita M, Ichisaka T (2007). Induction of pluripotent stem cells from adult human fibroblasts
by defined factors.. Cell.

[3] Yu J, Vodyanik MA, Smuga-Otto K, Antosiewicz-Bourget J, Frane JL (2007). Induced pluripotent stem cell lines derived from human somatic
cells.. Science.

[4] Yamanaka S (2009). A fresh look at iPS cells.. Cell.

[5] Yamanaka S, Blau HM (2010). Nuclear reprogramming to a pluripotent state by three
approaches.. Nature.

[6] Seki T, Yuasa S, Oda M, Egashira T, Yae K (2010). Generation of induced pluripotent stem cells from human
terminally differentiated circulating T cells.. Cell Stem Cell.

[7] Loh YH, Hartung O, Li H, Guo C, Sahalie JM (2010). Reprogramming of T cells from human peripheral
blood.. Cell Stem Cell.

[8] Staerk J, Dawlaty MM, Gao Q, Maetzel D, Hanna J (2010). Reprogramming of human peripheral blood cells to induced
pluripotent stem cells.. Cell Stem Cell.

[9] Baudin B, Bruneel A, Bosselut N, Vaubourdolle M (2007). A protocol for isolation and culture of human umbilical vein
endothelial cells.. Nat Protoc.

[10] Thomson JA, Itskovitz-Eldor J, Shapiro SS, Waknitz MA, Swiergiel JJ (1998). Embryonic stem cell lines derived from human
blastocysts.. Science.

[11] Ludwig TE, Bergendahl V, Levenstein ME, Yu J, Probasco MD (2006). Feeder-independent culture of human embryonic stem
cells.. Nat Methods.

[12] Ludwig TE, Levenstein ME, Jones JM, Berggren WT, Mitchen ER (2006). Derivation of human embryonic stem cells in defined
conditions.. Nat Biotechnol.

[13] Aasen T, Raya A, Barrero MJ, Garreta E, Consiglio A (2008). Efficient and rapid generation of induced pluripotent stem cells
from human keratinocytes.. Nat Biotechnol.

[14] Ruiz S, Brennand K, Panopoulos AD, Herrerías A, Gage FH (2010). High efficient generation of induced pluripotent stem cells from
astrocytes.. PLoS ONE.

[15] Liu GH, Barkho BZ, Ruiz S, Diep D, Qu J (2011). Recapitulation of premature ageing with iPSCs from
Hutchinson-Gilford progeria syndrome.. Nature.

[16] Maherali N, Hochedlinger K (2008). Guidelines and techniques for the generation of induced
pluripotent stem cells.. Cell Stem Cell.

[17] Maherali N, Hochedlinger K (2009). Tgfbeta signal inhibition cooperates in the induction of iPSCs
and replaces Sox2 and cMyc.. Curr Biol.

[18] Ichida JK, Blanchard J, Lam K, Son EY, Chung JE (2009). A small-molecule inhibitor of tgf-Beta signaling replaces sox2 in
reprogramming by inducing nanog.. Cell Stem Cell.

[19] Yoshida Y, Takahashi K, Okita K, Ichisaka T, Yamanaka S (2009). Hypoxia enhances the generation of induced pluripotent stem
cells.. Cell Stem Cell.

[20] Lagarkova MA, Shutova MV, Bogomazova AN, Vassina EM, Glazov EA (2010). Induction of pluripotency in human endothelial cells resets
epigenetic profile on genome scale.. Cell Cycle.

[21] Ho PJ, Yen ML, Lin JD, Chen LS, Hu HI (2010). Endogenous KLF4 expression in human fetal endothelial cells
allows for reprogramming to pluripotency with just OCT3/4 and
SOX2.. Brief Report. Arterioscler Thromb Vasc Biol.

[22] Zhu S, Li W, Zhou H, Wei W, Ambasudhan R (2010). Reprogramming of human primary somatic cells by OCT4 and chemical
compounds.. Cell Stem Cell.

[23] Giorgetti A, Montserrat N, Aasen T, Gonzalez F, Rodriguez-Piza I (2009). Generation of induced pluripotent stem cells from human cord
blood using OCT4 and SOX2.. Cell Stem Cell.

[24] Haase A, Olmer R, Schwanke K, Wunderlich S, Merkert S (2009). Generation of induced pluripotent stem cells from human cord
blood.. Cell Stem Cell.

[25] Miura K, Okada Y, Aoi T, Okada A, Takahashi K (2009). Variation in the safety of induced pluripotent stem cell
lines.. Nat Biotechnol.

[26] Taylor CJ, Bolton EM, Pocock S, Sharples LD, Pedersen RA (2005). Banking on human embryonic stem cells: estimating the number of
donor cell lines needed for HLA matching.. Lancet.

[27] Nakatsuji N, Nakajima F, Tokunaga K (2008). HLA-haplotype banking and iPS cells.. Nat Biotechnol.

